# Widespread detections of neonicotinoid contaminants in central Wisconsin groundwater

**DOI:** 10.1371/journal.pone.0201753

**Published:** 2018-10-03

**Authors:** Benjamin Z. Bradford, Anders S. Huseth, Russell L. Groves

**Affiliations:** 1 Department of Entomology, University of Wisconsin-Madison, Madison, WI, United States of America; 2 Department of Entomology, North Carolina State University, Raleigh, NC, United States of America; University of Pittsburgh, UNITED STATES

## Abstract

Neonicotinoids are a popular and widely-used class of insecticides whose heavy usage rates and purported negative impacts on bees and other beneficial insects has led to questions about their mobility and accumulation in the environment. Neonicotinoid compounds are currently registered for over 140 different crop uses in the United States, with commercial growers continuing to rely heavily on neonicotinoid insecticides for the control of key insect pests through a combination of in-ground and foliar applications. In 2008, the Wisconsin Department of Agriculture, Trade and Consumer Protection (DATCP) began testing for neonicotinoids in groundwater test wells in the state, reporting detections of one or more neonicotinoids in dozens of shallow groundwater test wells. In 2011, similar detection levels were confirmed in several high-capacity overhead center-pivot irrigation systems in central Wisconsin. The current study was initiated to investigate the spatial extent and magnitude of neonicotinoid contamination in groundwater in and around areas of irrigated commercial agriculture in central Wisconsin. From 2013–2015 a total of 317 samples were collected from 91 unique high-capacity irrigation wells and tested for the presence of thiamethoxam (TMX), a neonicotinoid, using enzyme-linked immunosorbent assays. 67% of all samples were positive for TMX at a concentration above the analytical limit of quantification (0.05 μg/L) and 78% of all wells tested positive at least once. Mean detection was 0.28 μg/L, with a maximum detection of 1.67 μg/L. Five wells had at least one detection exceeding 1.00 μg/L. Furthermore, an analysis of the spatial structure of these well detects suggests that contamination profiles vary across the landscape, with differences in mean detection levels observed from landscape (25 km), to farm (5 km), to individual well (500 m) scales. We also provide an update of DATCP’s neonicotinoid monitoring in Wisconsin’s shallow groundwater test wells and private potable wells for the years 2011–2017.

## Introduction

Neonicotinoids are a popular and widely-used class of insecticides whose water-soluble nature and 20-year usage history has led to questions about their potential to accumulate in the environment and harm local ecosystems [1–6]. When first registered in the United States in 1995, these compounds promised increased efficacy, long-lasting systemic activity, lower application rates, low vertebrate toxicity, and reduced environmental persistence, all of which contributed to the rapid adoption and widespread use of this class of insecticides, which now account for over 25% of the entire global pesticide market [7]. Over 6.7 million pounds of neonicotinoid insecticides are now applied annually on 140 different crops in the United States, with the three most popular compounds, imidacloprid (IMD), clothianidin (CLO), and thiamethoxam (TMX) making up over 90% of agricultural usage nationally [7,8].

Most neonicotinoids are registered for application as seed treatments, foliar sprays, and in-furrow soil drenches, with seed treatments and soil applications constituting 60% of agricultural neonicotinoid usage [7]. Seed and soil application methods are of particular environmental concern because uptake rates of applied active ingredients have been reported as 2–5% in cotton, eggplant, potato, and rice, and up to 20% in maize, meaning that in excess of 80% of applied active ingredients remain in field soils potentially resulting in off-site movement and environmental contamination [9]. Neonicotinoids compounds have reported soil half-lives measured in months to years depending on conditions such as temperature, depth, and microbial activity (IMD: 100–1230 days; CLO: 148–7000 days; TMX: 3.4–1000 days) [4]. The risk that these long field persistence times will translate into off-site movement of neonicotinoid compounds is further increased by the high water solubility of the major neonicotinoid compounds: IMD = 610 mg/L; CLO = 340 mg/L; and TMX = 4100 mg/L [4]. Indeed, laboratory and field studies have demonstrated a high risk of leaching associated with soil and seed applications of neonicotinoid insecticides [2,10–12].

Emerging concern about neonicotinoid contamination has motivated the development of ecosystem- and regional-scale water quality surveys [5,13–17]. Conservation groups have also raised calls for neonicotinoids to be banned or phased out due to the substantial ecological risks their continued use may pose [18,19]. Neonicotinoid residues have now been documented in a variety of locations in and around agricultural fields including in dusts exhausted during drilling of treated seed (68–15,000 mg/Kg) [20], pollen (up to 51 μg/L) and nectar (up to 8.6 μg/L) of treated plants [1,21], plant guttation fluid (10–200 mg/L) [22], soil (1–100 mg/Kg) [1], puddles (up to 63 μg/L) [23], and surface water systems (up to 225 μg/L) [6,18,19]. Recent reports of neonicotinoid detections in surface waters across the United States have been reviewed in [18,24]: maximum IMD detections were reported as 3.29 μg/L in California [14], 6.90 μg/L in Massachusetts [25], 9.00 μg/L in South Carolina [26], and 25 μg/L in Maryland [27]. One study reported CLO detections up to 0.257 μg/L in Iowa [16]. TMX detections have been reported up to 2.49 μg/L in South Dakota [18], 8.93 μg/L in Wisconsin [2], and 225 μg/L in the playa wetlands of the Southern High Plains of the United States [28]. Recent surveys in the US Midwest have also indicated that neonicotinoid contaminants can be found year-round in 10 different tributaries of the Great Lakes spanning six states [15].

Neonicotinoid detections in surface water systems at these concentrations are cause for alarm as aquatic invertebrates are key members of many freshwater ecosystems and some species are extremely sensitive to neonicotinoid insecticides, with acute toxicity endpoints reported down to 1 μg/L and chronic toxicity endpoints reported down to 0.1 μg/L [6]. In that paper Morrissey *et al*. suggest an ecological threshold for neonicotinoids be established at 0.2 μg/L long-term acute and 0.035 μg/L long-term chronic exposure limits. Similar aquatic invertebrate benchmarks of 0.385 μg/L acute exposure and 0.01 μg/L chronic exposure have been established by the US Environmental Protection Agency (EPA) as part of their registration review of IMD [29]. EPA benchmarks also exist for the neonicotinoids CLO (11 μg/L acute, 1.1 μg/L chronic), TMX (17.5 μg/L acute, no chronic benchmark listed), acetamiprid (10.5 μg/L acute, 2.1 μg/L chronic), and thiacloprid (18.9 μg/L acute, 0.97 μg/L chronic) (US EPA, available https://www.epa.gov/pesticide-science-and-assessing-pesticide-risks/aquatic-life-benchmarks-and-ecological-risk). Currently these benchmarks are only advisory and any detections exceeding these benchmarks will not result in any regulatory action but are helpful in evaluating the potential ecological effects of any environmental neonicotinoid detections in surface and groundwater.

In Wisconsin, groundwater monitoring efforts have been conducted by the state department of Agriculture, Trade and Consumer Protection, Environmental Quality Section (DATCP). DATCP regularly tests for select contaminants in private potable wells and groundwater monitoring wells as part of its mission of monitoring and protecting water quality in the state. These samples encompass several ongoing survey efforts, including new private potable wells pending certification, private potable wells flagged for resampling due to past detections of certain chemicals exceeding enforcement standards (such as nitrates and the herbicide atrazine), and from static groundwater monitoring wells established to monitor shallow groundwater for agricultural contaminants in locations deemed at elevated risk of such contamination. In 2008, DATCP added tests for select neonicotinoids (initially only TMX, later IMD, CLO, and others) as a part of this groundwater monitoring effort in response to significant public concern among rural communities about the rapidly expanding use of this new class of insecticides and their potential for accumulation in groundwater resources [30,31]. These surveys revealed concentrations of one or more neonicotinoid compounds in dozens of test wells, with most detections occurring in the Central Sands and Lower Wisconsin River Valley (LWRV) agroecosystems. In addition, similar concentrations of neonicotinoid active ingredients were also detected in water drawn from a small number of high-capacity overhead center-pivot irrigation systems (defined by the Wisconsin Department of Natural Resources as capable of pumping more than 380,000 L of water per day) used to water potatoes and processing vegetables [2].

The frequency of neonicotinoid detections specifically in the Central Sands and LWRV agroecosystems suggested that further study of this area was warranted. A significant fraction of irrigated potato and processing vegetable production in Wisconsin occurs in the Central Sands and LWRV and neonicotinoid insecticides are frequently employed as crop protectants by local growers. In addition, the hydrology of these regions is characterized by sandy, fast-draining soils, and shallow, unconfined aquifers that have been identified as at an elevated risk of contamination according to the Wisconsin Groundwater Contamination Model (*Ecological Landscapes of Wisconsin—Map S16*, Wisconsin Department of Natural Resources, available: http://dnr.wi.gov/topic/landscapes/). The present study was initiated to assess the magnitude, spatial extent, and temporal dynamics of neonicotinoid contamination in groundwater in these regions at a higher spatial and temporal resolution than existed within the monitoring data available from state agencies. To assess the extent of contamination we perform a structured, multi-year study of neonicotinoid contamination in high-capacity irrigation wells distributed throughout the Central Sands and Lower Wisconsin River Valley agroecosystems in Wisconsin. Irrigation wells provide both a broad spatial sampling scale (landscape to state), can be sampled repeatedly during growing seasons, and draw groundwater from deeper than the static test wells sampled by DATCP, potentially revealing the extent to which contaminants have permeated the underlying aquifers. In addition to our high-capacity well observations, we also present the results of neonicotinoid monitoring in shallow groundwater test wells and private potable wells conducted by the Wisconsin DATCP from 2011 through 2017 in the same geographic area.

## Materials and methods

### Ethics statement

No ethics approval was required to conduct this study. All private landowners who participated in this study and granted access to their irrigation systems have done so with assurances that they would remain anonymous and so GPS coordinates and high-capacity well identification numbers cannot be shared as part of that confidentiality agreement. No special permissions were required to share data generated by the Wisconsin Department of Agriculture, Trade, and Consumer Protection and presented herein. This study did not involve any threatened or endangered species.

### State-run water quality surveys

A database of neonicotinoid detections and non-detections for field-edge and private potable wells was acquired from the Wisconsin Department of Agriculture, Trade and Consumer Protection (DATCP), with coverage from 2008 through 2017 (Jeff Postle and Rick Graham, DATCP, personal communication). Neonicotinoid concentrations in private potable and groundwater monitoring well samples reported by the Wisconsin DATCP were determined by liquid chromatography-tandem mass spectrometry at the DATCP Bureau of Laboratory Services. Assays were performed for IMD, CLO, and TMX in all years; acetamiprid, dinotefuran, and thiacloprid were also included beginning in 2016. Limits of detection for all neonicotinoid compounds were 0.2 μg/L from 2011 through September 2014 and 0.05 μg/L thereafter. Within this dataset, multiple well identifiers referred to identical physical locations, so for the purposes of our analysis we grouped well ID’s that occur within 0.0001 decimal degrees of latitude or longitude (approximately 8–12 meters) and refer to these as ‘well sites’. Here we report detections from 2011–2017; results from 2008–2010 have been previously discussed [2].

### High-capacity well study

A significant fraction of irrigated potato and processing vegetable production in Wisconsin occurs in the regions known as the Central Sands and the Lower Wisconsin River Valley (LWRV). From these regions, one vegetable grower located in the LWRV, five growers from the Central Sands, and the Hancock Agricultural Research Station (operated by the University of Wisconsin and located within the Central Sands near Hancock, WI) agreed to participate in this study, though not all groups participated each year. Initially, each grower was asked to identify up to 10 high capacity wells for periodic water collections from among their managed fields. In subsequent years, growers were asked to continue sampling the same wells, though this was not always possible, so other available wells were substituted at each farm location. For the purposes of describing the spatial distribution of wells and any detected contaminants, wells were first grouped by farm (indicated by letter codes A-G), then by geographic regions of roughly similar size. Both the Plover and Moraine regions lie within the Central Sands but have been separated into two regions here to approximately match the size of the Lower Wisconsin region. Farms are approximately 5-10km in size, while regions are approximately 25-40km, except for the LWRV region, where all wells sampled there were under the management of a single farm operation. For each farm, the number of sampled wells each year, mean well depth, water table depth, and distance between wells are reported in **Table 1**.

**Table 1:** Physical and spatial characteristics of high-capacity wells sampled in this study. Well depth and water table depth averages calculated from a database of high-capacity wells provided by the Wisconsin Department of Natural Resources. Well spacing calculated from the linear distance between a well and its closest neighbor within the specified group.

Farm	Region	No. of wells			Well characteristics						Well spacing (km)		
		2013	2014	2015	Inlet depth (m)			Depth to water (m)			Min	Mean	Max
**A**	Plover	10	10	11	21.34	±	4.97	7.70	±	3.89	0.35	0.62	1.04
**B**	Plover	-	-	6	20.27	±	4.29	3.38	±	1.98	1.30	2.45	4.58
**C**	Moraine	10	10	12	23.57	±	5.10	3.07	±	1.27	0.45	1.13	3.24
**D**	Moraine	6	6	-	28.96	±	3.70	5.89	±	2.09	0.27	0.45	0.62
**E**	Moraine	5	9	9	36.90	±	8.37	12.52	±	5.38	0.35	0.72	1.44
**F**	Moraine	8	8	8	37.57	±	6.52	7.58	±	3.20	0.64	0.84	1.13
**G**	LWRV	9	10	10	30.88	±	6.74	3.68	±	1.76	0.10	1.08	4.78
		**48**	**53**	**56**	**29.45**	**±**	**8.57**	**5.89**	**±**	**4.39**	**0.10**	**0.96**	**4.78**

In 2013, each high-capacity well was sampled once in late August or early September. In 2014, wells were sampled once approximately in the middle of the growing season (June/July) and once towards the end of the growing season (August/September). For the 2015 field season, wells were sampled approximately monthly from May-September, with a few wells sampled into October, to better reveal any seasonal trends in TMX detections. In addition, one well on three farms was identified for weekly rather than monthly sampling to further increase the temporal resolution at those locations. As samples were collected from operating irrigation wells, the timing of sample collection was necessarily constrained to the typical vegetable growing season in the area (May–October).

Water samples were collected from wells that had been operating at full capacity for at least fifteen minutes prior to sample collection to ensure that water samples were properly representative of the groundwater surrounding the well inlet. Most water sampling was performed by cooperating growers who adhered to this protocol and kept water samples refrigerated until collection and analysis. During the 2013 and 2014 field seasons, samples were collected in triple-rinsed, 250 mL amber glass bottles; in the 2015 field season, the amber glass bottles were substituted with Nalgene amber HDPE bottles. Each primary water sample consisted of one 250 mL collection, from which three 1.5 mL aliquots were taken and stored frozen (-4°C) in 2.0 mL microfuge tubes for later analysis. We chose to assay only for TMX as a sentinel neonicotinoid in our water samples to reduce costs and because it is a common insecticide applied in commercial vegetable production in our survey area.

TMX concentrations were determined using commercially available, 96-well competitive enzyme-linked immunosorbent assay (ELISA) kits (Thiamethoxam HS Plate Kit, Cat. # 20–0102, Beacon Analytical Systems, Saco, ME). TMX concentration was quantified using an optical plate reader (VersaMax tunable microplate reader, Molecular Devices, Sunnyvale, CA) and the accompanying Softmax Pro software. Each plate was standardized against a four-parameter logistic curve generated from the four standards provided in the kit (0, 0.05, 0.30 and 2.00 μg/L) run alongside well water samples. All standards and samples were run in triplicate and results averaged to minimize plate-level error. The limit of quantification for the assays is 0.05 μg/L according to the manufacturer. Values above this limit are referred to in this text as “detections” or “detects”. No samples were found to exceed 2.0 μg/L, so no sample dilution was necessary for accurate determination of concentrations.

### Data analysis

All statistical analyses were performed in R, version 3.4.1 [32]. Functions used in data analysis are available in the base distribution package unless otherwise noted. TMX concentration values were log transformed (log_10_[x+0.01]) prior to analysis to satisfy assumptions of normality. Summary statistics specifically referencing “detections” do not include any values falling below the analytical limit of quantification. For certain figures and analyses where the inclusion of all kit results was warranted, values below the limit of quantification were left as determined by the kit rather than being re-coded as zeroes. Variance component estimates were calculated from a random effects model that included the three spatial (*region*, *farm*, *well*) and three temporal factors (*year*, *season*, *month*) previously introduced. A fitted random effects model was generated using the function *lme* [33]. Variance component analysis of this fitted object was generated using the function *varcomp* [34]. Spatial factors were nested sequentially from larger to smaller scale, with temporal factors further nested within these spatial factors.

## Results

### Wisconsin DATCP field-edge and private potable well surveys

**Fig 1:**
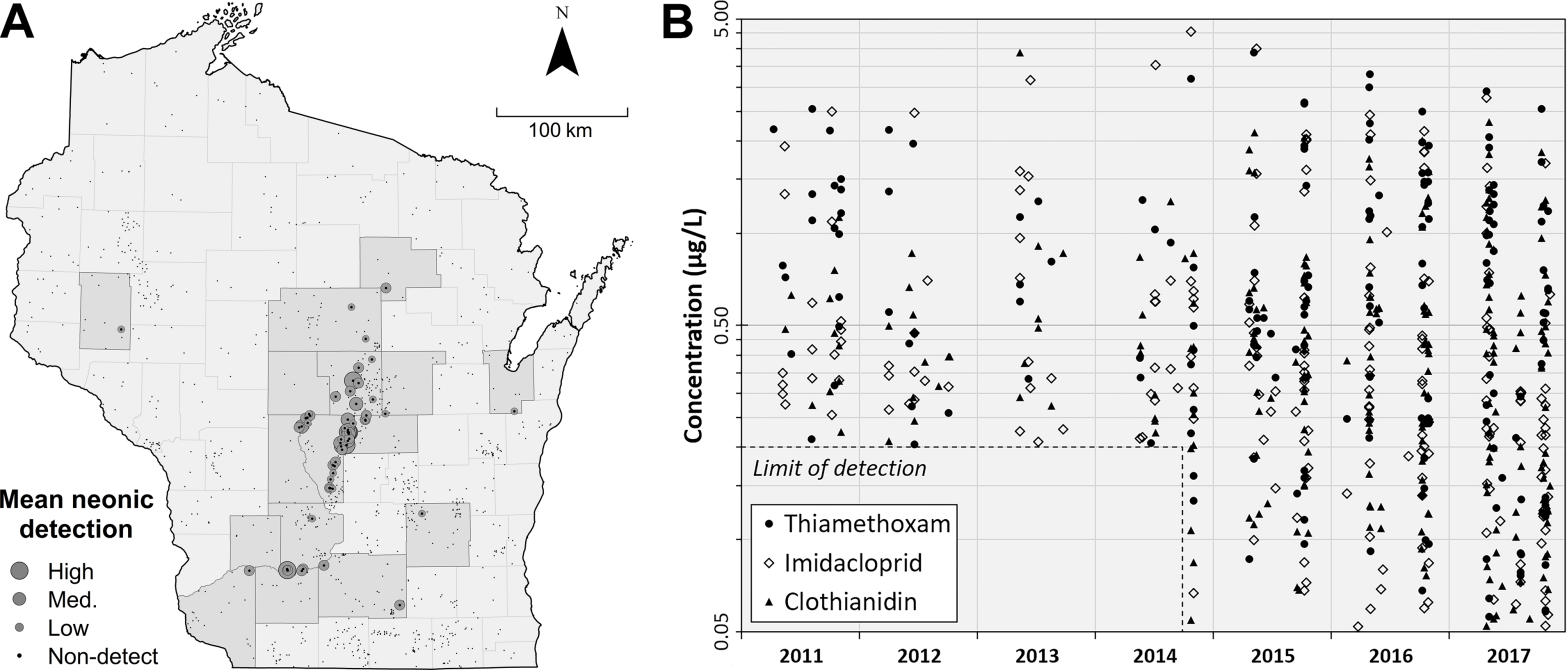
Distribution of neonicotinoid detections in private potable wells and groundwater test wells in central Wisconsin, 2011–2017. Map in (**A**) illustrates locations of neonicotinoid detections in monitoring wells and private potable wells within the state of Wisconsin as reported by the Wisconsin Department of Agriculture, Trade, and Consumer Protection, Environmental Quality Section, 2011–2017. Positive detections are illustrated by grey circles, with a larger diameter reflecting higher average total neonicotinoid detection at that particular location (range 0.01–3.93 μg/L). Wells tested but returning no positive detections over the surveillance interval are indicated as dots. Counties with at least one positive detection are shaded in grey. Chart (**B**) shows all positive detections for thiamethoxam, imidacloprid, and clothianidin over time, as well as the analytical limit of detection, which was initially 0.20 μg/L and later 0.05 μg/L.

The Wisconsin DATCP has been testing select field-edge groundwater monitoring wells and private potable wells for neonicotinoid compounds since 2008. These samples were collected as part of the agency’s broader agrichemical contamination monitoring program and were also screened for a number of other contaminants, but here we cover only the results of their neonicotinoid assays. The location of all wells and location and magnitude of all positive neonicotinoid detections is illustrated in **Fig 1A**. Individual detections for TMX, IMD, and CLO from 2011–2017 as well as the analytical limit of quantification is illustrated in **Fig 1B**.

From 2011 through 2017, 28 of the 53 monitoring well sites tested positive for at least one neonicotinoid, with 5 wells testing positive for two neonicotinoids, and 14 wells testing positive for IMD, CLO, and TMX during this seven-year period (**Table 2**). Of the 527 total samples collected from monitoring wells, 150 (28%) tested positive for TMX, 162 (31%) tested positive for IMD, and 194 (37%) testing positive for CLO. Mean TMX detection was 0.90 μg/L, with a maximum detection of 3.89 μg/L recorded in Adams Co. in 2015. Mean IMD detection was 0.61 μg/L, with a maximum of 4.54 μg/L recorded in Waushara Co. in 2014. Mean CLO detection was 0.503 μg/L, with a maximum of 2.30 μg/L recorded in Dane Co. in 2017. In 2016 and 2017, DATCP also tested all monitoring well samples for the three less common neonicotinoids acetamiprid, dinotefuran, and thiacloprid. No monitoring well samples were positive for these three compounds at concentrations above the detection limit of 0.05 μg/L.

**Table 2:** Neonicotinoid detections, WI-DATCP groundwater monitoring program, 2011–2017. Summary of neonicotinoid detections in Wisconsin groundwater monitoring wells and private potable well samples, 2011–2017. Wells occurring within approximately 8-12m were grouped into “unique locations” for this summary. Concentrations were determined by liquid chromatography-tandem mass spectrometry at the Bureau of Laboratory Services. Limits of detection for neonicotinoid compounds were 0.20 μg/L from 2011 through September 2014 and 0.05 μg/L thereafter. Data courtesy Wisconsin Department of Agriculture, Trade, and Consumer Protection, Environmental Quality Section.

Well Type	Year	Unique locs	No well sites with specified number of contaminants					Total samples	N positive	% positive	Thiamethoxam (μg/L)				Imidacloprid (μg/L)				Clothianidin (μg/L)			
			None	1+	1	2	3				Pos.	Freq.	Mean	Max	Pos.	Freq.	Mean	Max	Pos.	Freq.	Mean	Max
***Monitoring well***																						
	2011	53	29	13	7	4	2	96	28	29%	17	18%	1.12	2.55	17	18%	0.70	2.50	10	10%	0.52	1.13
	2012	43	28	11	5	5	1	53	13	25%	8	15%	0.93	2.17	8	15%	0.61	2.47	7	13%	0.47	0.86
	2013	37	27	7	6	1	0	37	7	19%	1	3%	1.27	1.27	3	8%	1.23	3.16	4	11%	0.58	0.91
	2014	42	22	11	4	2	5	52	21	40%	12	23%	0.67	3.19	13	25%	0.97	4.54	16	31%	0.37	0.86
	2015	41	13	15	2	4	9	72	42	58%	26	36%	0.88	3.89	31	43%	0.71	4.00	38	53%	0.52	2.05
	2016	51	13	15	2	4	9	91	56	62%	38	42%	1.07	3.31	39	43%	0.60	2.43	49	54%	0.54	2.01
	2017	38	14	24	8	4	12	126	82	65%	48	38%	0.74	2.90	51	40%	0.39	2.77	70	56%	0.49	2.30
	**Total**	**53**	**25**	28	**9**	**5**	**14**	**527**	**249**	**47%**	**150**	**28%**	**0.90**	**3.89**	**162**	**31%**	**0.61**	**4.54**	**194**	**37%**	**0.50**	**2.30**
***Private potable***																						
	2011	146	144	1	0	1	0	146	1	1%	1	1%	1.43	1.43	0	0%	-	-	1	1%	0.47	0.47
	2012	113	106	5	3	1	1	116	5	4%	1	1%	0.26	0.26	3	3%	0.45	0.70	4	3%	0.37	0.40
	2013	121	111	10	4	4	2	122	10	8%	5	4%	0.71	1.13	9	7%	0.82	1.59	4	3%	1.33	3.88
	2014	113	99	10	6	2	2	116	10	9%	3	3%	0.72	1.03	8	7%	0.44	0.70	5	4%	0.58	1.27
	2015	169	157	11	5	1	5	174	12	7%	7	4%	0.54	1.13	8	5%	0.38	1.06	10	6%	0.54	2.13
	2016	495	485	9	6	1	2	497	9	2%	3	1%	0.26	0.29	7	1%	0.27	1.01	4	1%	0.18	0.38
	2017	142	130	12	7	4	1	142	12	8%	4	3%	0.15	0.22	5	4%	0.29	0.63	9	6%	0.20	0.63
	**Total**	**1120**	**1069**	51	**27**	**13**	**11**	**1313**	**59**	**4%**	**24**	**2%**	**0.52**	**1.43**	**40**	**3%**	**0.47**	**1.59**	**37**	**3%**	**0.49**	**3.88**
***All sources***																						
	**Total**	**1173**	**1094**	79	**36**	**18**	**25**	**1840**	**308**	**17%**	**174**	**9%**	**0.85**	**3.89**	**202**	**11%**	**0.58**	**4.54**	**231**	**13%**	**0.50**	**3.88**

Neonicotinoid compounds were detected with significantly less frequency among private potable well samples as these private wells are distributed throughout the state, whereas monitoring wells have been specifically established to monitor agricultural chemical intrusion into aquifers in areas where past contamination has been detected or where the risk of such contamination was considered elevated. During the 2011–2017 period, WI-DATCP collected and tested 1313 samples from 1120 individual private potable wells. 51 wells tested positive for at least one neonicotinoid compound, with 27 wells positive for one, 13 wells positive for two, and 11 wells positive for all three major neonicotinoids. TMX was detected in 59 samples (4%), with a mean of 0.52 μg/L and a maximum of 1.43 μg/L, recorded in Sauk Co. in 2011. IMD was detected in 40 samples (3%), with a mean of 0.47 μg/L and a maximum of 1.59 μg/L recorded in Waushara Co. in 2013. CLO was detected in 37 samples (3%), with a mean detection of 0.49 μg/L and a maximum of 3.88 μg/L recorded in Waushara Co. in 2013. All samples collected from private potable wells in 2016 and 2017 were also tested for the less common neonicotinoids acetamiprid, dinotefuran, and thiacloprid. One sample, from Junea Co. in 2017, tested positive for dinotefuran at 0.15 μg/L.

### Thiamethoxam detections in high-capacity irrigation wells

**Fig 2:**
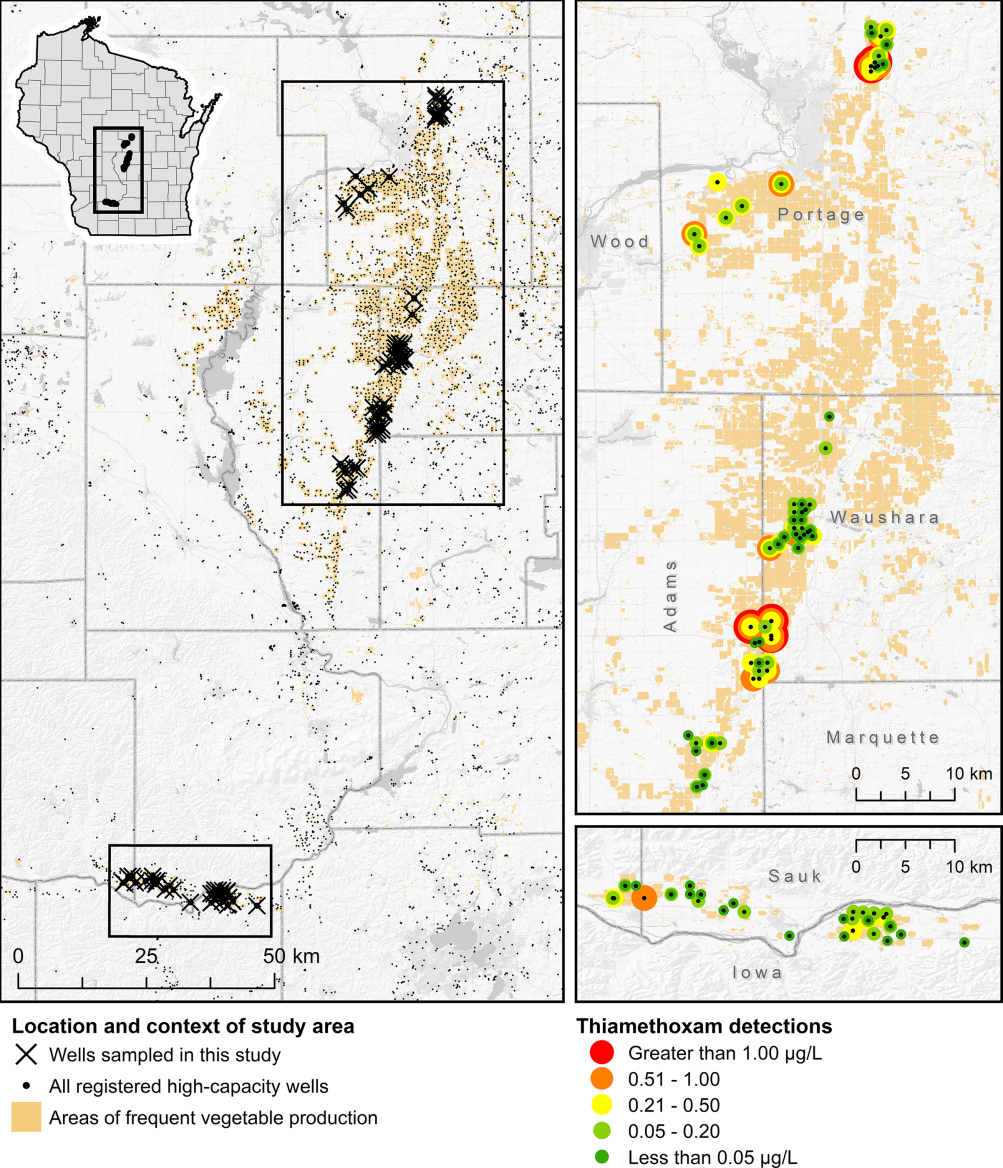
Locations of high-capacity wells and results of TMX assays. On left, locations of all registered high-capacity wells in the area and the subset of those wells sampled in this study. Beige color reflects areas of frequent vegetable production (https://nassgeodata.gmu.edu/CropScape/). Study wells are grouped by letter codes reflecting the farm that operated that field and well during the sampling year. Location data for all registered high-capacity wells courtesy Wisconsin Department of Natural Resources. TMX concentrations below the analytical limit of quantification of 0.05 μg/L are considered non-detections.

Over the entire three-year period of these investigations, 317 total samples were collected from 91 unique high-capacity irrigation wells (**Table 3, Fig 2**). Overall 78% of wells tested positive at least once for TMX at a concentration above the limit of quantification (0.05 μg/L) of the ELISA kits, with a maximum detection of 1.69 μg/L and a mean detection of 0.28 ± 0.29 μg/L. In 2013, 48 unique high-capacity wells were sampled once at the end of the vegetable growing season (late August to early September). In this survey year, 81% (39/48) of wells sampled returned a positive TMX detection (concentration > 0.05 μg/L), with a mean positive detection of 0.28 ± 0.28 μg/L and a maximum detection of 1.56 μg/L. In 2014, 79 samples were collected from 53 wells, of which 35 independent wells (66%) returned at least one positive detection. Mean detection was 0.26 ± 0.27 μg/L with a maximum detection of 1.21 μg/L. In 2014, the second year of our study, we divided sampling into mid-season (mid Jun–early Aug) and late-season (late Aug—Sept) portions of the growing season to better understand patterns of detection during the growing season. The detection rate of mid-season samples was 64% (34/53) with a maximum of 1.06 μg/L and mean of 0.25 ± 0.26 μg/L; the late-season detection rate was 73% (19/26) with a maximum of 1.21 μg/L and a mean of 0.28 ± 0.29 μg/L. In 2015, 56 wells were sampled approximately monthly from May through September (several wells were sampled into October) for a total of 190 samples. Three of these wells were sampled weekly rather than monthly during this same period. Of the 56 total wells sampled in 2015, 40 (71%) had at least one TMX detection above the limit of quantification. Maximum detection in 2015 was 1.69 μg/L, and mean detection was 0.29 ± 0.30 μg/L. Early season (May) detections averaged 0.32 ± 0.27 μg/L with a maximum of 0.89 μg/L and a detection rate of 62% (34/55 samples), mid-season (June-July) detections averaged 0.32 ± 0.37 μg/L with a maximum of 1.69 μg/L and a detection rate of 65% (54/83 samples), and late-season detections averaged 0.21 ± 0.17 μg/L with a maximum of 0.77 μg/L and a detection rate of 63% (33/52 samples).

**Table 3:** TMX detections in high-capacity irrigation wells, 2013–2015.

Year	Timing	Months	Wells			Samples			Detections			
			n	Pos.*	% Pos.	n	Pos.*	% Pos.	Max	Mean	±	SD
**2013**	Late	Aug/Sep	48	39	81%	48	39	81%	1.56	0.28	±	0.28
**2014**	Mid	Jun/Jul	53	34	64%	53	25	47%	1.06	0.25	±	0.26
	Late	Aug/Sep	26	19	73%	26	19	73%	1.21	0.28	±	0.29
		*Year Total*	53	35	66%	79	53	67%	1.21	0.26	±	0.27
**2015**	Early	May	40	27	68%	55	34	62%	0.89	0.32	±	0.27
	Mid	Jun/Jul	52	35	67%	83	54	65%	1.69	0.32	±	0.38
	Late	Aug-Oct	40	25	63%	52	33	63%	0.77	0.21	±	0.17
		*Year Total*	56	40	71%	190	121	64%	1.69	0.29	±	0.30
*Grand total*			**91**	**71**	**78%**	**317**	**213**	**67%**	**1.69**	**0.28**	**±**	**0.29**

*TMX concentrations > 0.05 μg/L are considered positive detections.

### Spatial and temporal variation in detections

**Fig 3:**
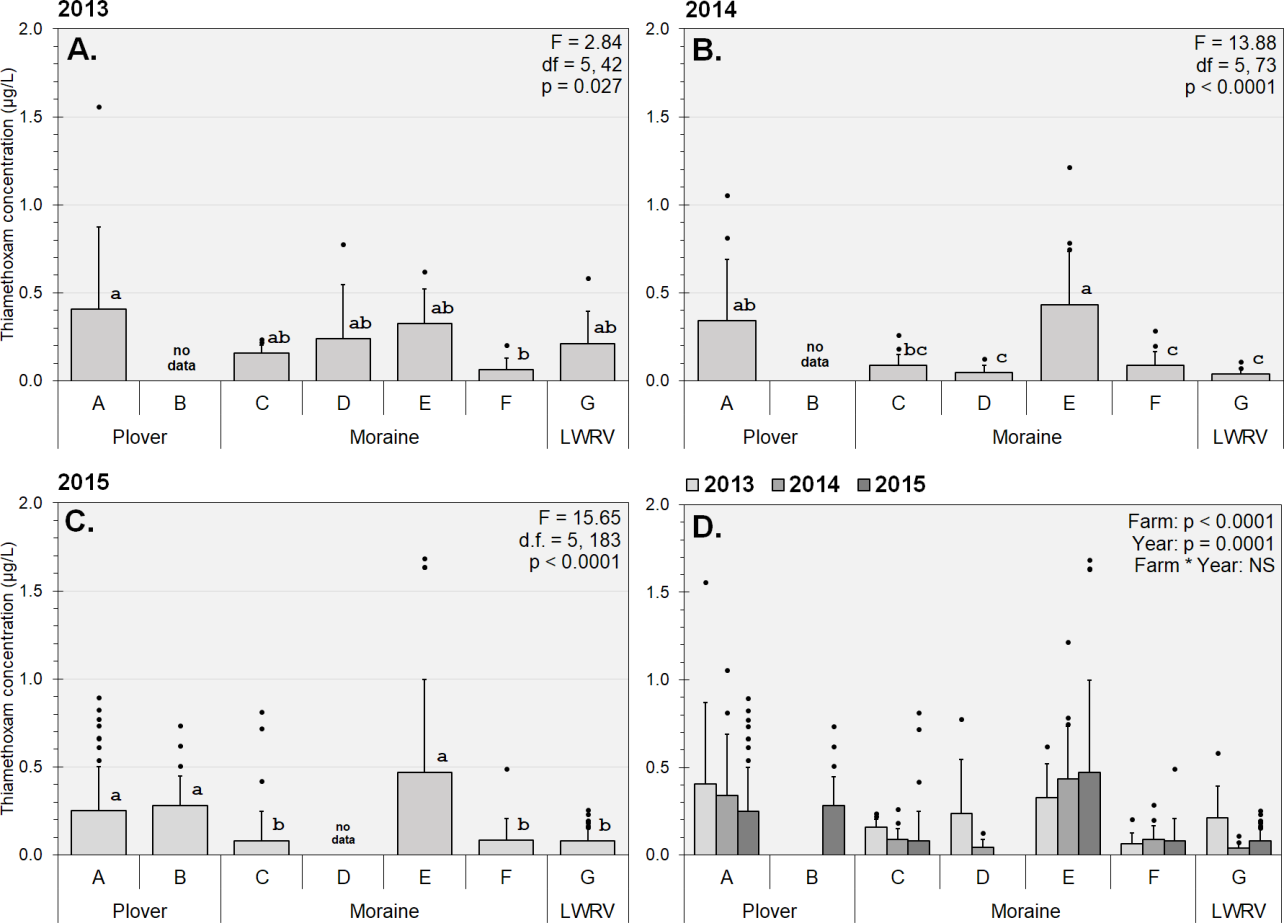
Mean TMX concentration in high-capacity wells by farm and year. Shaded bars represent average concentration (including non-detections) on farm, with standard deviation error bars. Black circles represent detections greater than one standard deviation above mean. For panels A-C, means separations are indicated by lower-case letter codes (Tukey’s HSD; α = 0.05). Panel D illustrates a significant farm (*F* = 19.296; df = (6,271); *P <* .0001) and year (*F* = 6.783, df = (2,271); *P =* .0013) main effect, with no significant interaction term.

Within each year of this study, we observed significant differences in mean TMX concentrations in high-capacity wells between farms (**Fig 3**). Wells on farms A, B, and E consistently possessed the highest average neonicotinoid concentrations across years, though mean concentration on most farms (except E) fell slightly each year. Note that Farm B only participated in 2015, and Farm D did not. While consistently higher detections on certain farms is grounds for further investigation, we cannot be sure which of the many human and physical factors that vary between farm locations may be responsible for these higher detections. However, farms C and D do overlap in space and TMX detections did not differ significantly between these farms for any year in which they were both sampled. Unless neonicotinoid usage rates differed significantly between farms, this does seem to suggest that physical and landscape characteristics may be playing an important role in the average contamination values in wells on these farms.

### Weekly sampling of select wells

**Fig 4:**
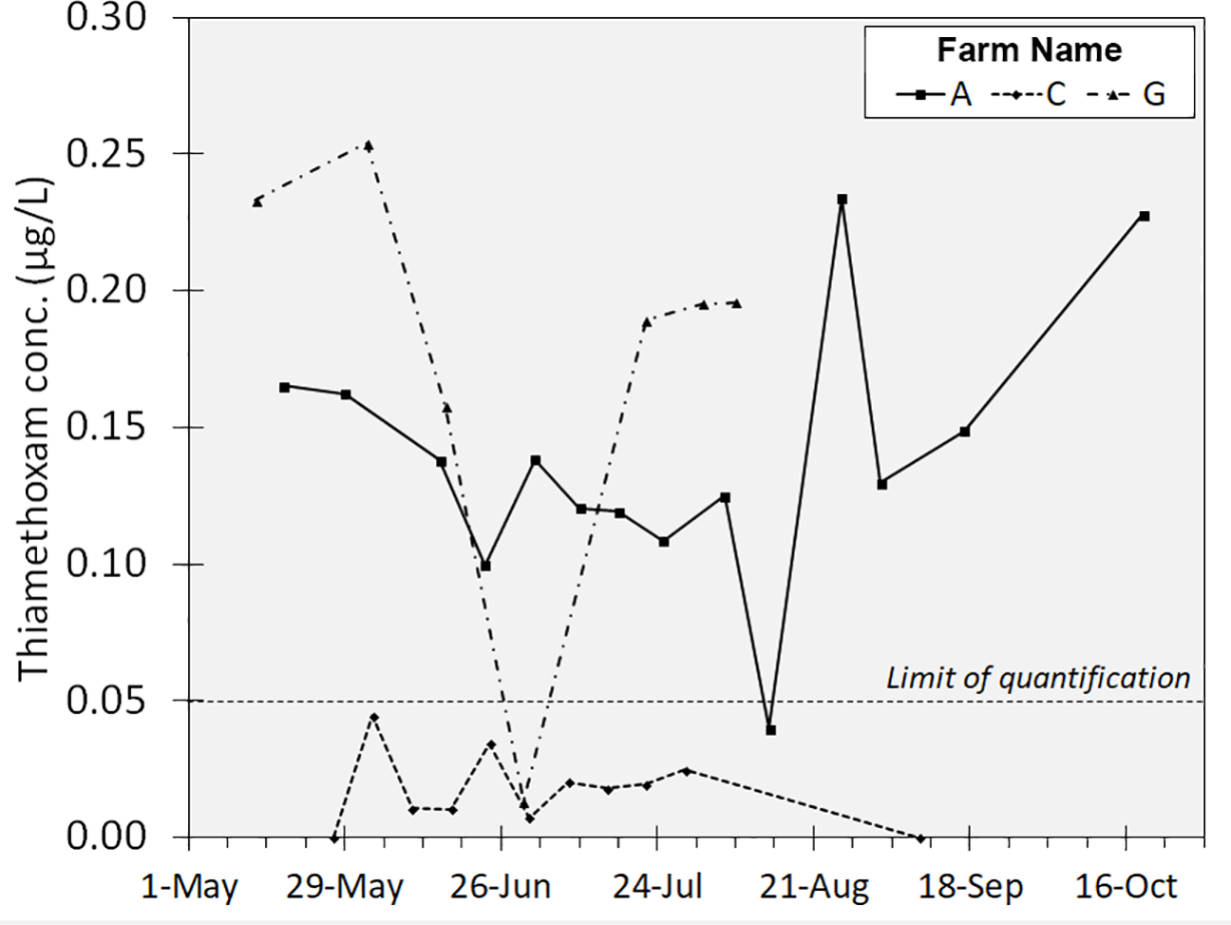
Weekly sampling of high-capacity wells on select farms, 2015. TMX concentrations in water sampled approximately weekly from one well on each of three farms in 2015. 14 samples were collected from farm A, 11 samples from farm C, and 7 samples from farm G.

During 2015, our collaborators on farms A, C, and G agreed to sample one of their wells on a weekly rather than monthly basis to increase the temporal resolution of our sampling at select well locations. While most samples from Farms A and G were positive detections, no sample from Farm G was above the limit of quantification (**Fig 4**). To evaluate the significance of farm and sampling week as explanatory factors of TMX concentration in these wells, a standard least-squares regression model including *farm*, farm*week, *week*, and week*week (starting May 1) was constructed. Results showed the *farm* main effect as a significant factor (F = 30.5, df = 2,25, P < .0001), as expected, considering the differences in mean concentration between farms noted previously. However, the *week* main effect term (F = 0.42 df = 1,25, P>.05), farm*week interaction term (F = 0.12, df = 2,25, P>.05), and week*week polynomial term (F = 1.94, df = 1,25, P>.05) were not significant. Despite the lack of statistical significance of the temporal terms in this model, it is clear that concentrations vary from week to week at each well location, though not necessarily in a predictable way. More frequent sampling, taking several repeated samples and averaging the results, using polar organic chemical integrative samplers (POCIS), or sampling other wells with higher average detections could help improve these results in the future. Prior work has shown increased mobility of soil- and seed-applied neonicotinoids in potato fields later in the season after vine-kill and crop harvest [2], so looking at relationships with previous year neonicotinoid inputs might yield promising results.

### Variance component analysis

One of the primary aims of this study was to determine the spatial scale at which differences in contamination levels can be observed. In order to quantify the relative importance of each of spatial scale (*region*, ~35 km; *farm*, ~10 km; and *well*, ~1 km), we performed a variance component analysis on a nested random effects model containing these three spatial factors to determine the relative contribution of each factor in partially explaining the observed variation in TMX concentrations. While *region* and *farm* explained 11.4% and 13.6% of variance, respectively, the single most significant explanatory factor was *well*, explaining 51.2% of variance in TMX concentrations. The remaining 23.8% of variance is not explained by these factors. These results suggest that while differences exist in average TMX concentration and detection frequency between regions and farms, the most significant spatial factor in explaining both response variables is the individual well (400–800 m typical separation between wells).

**Fig 5:**
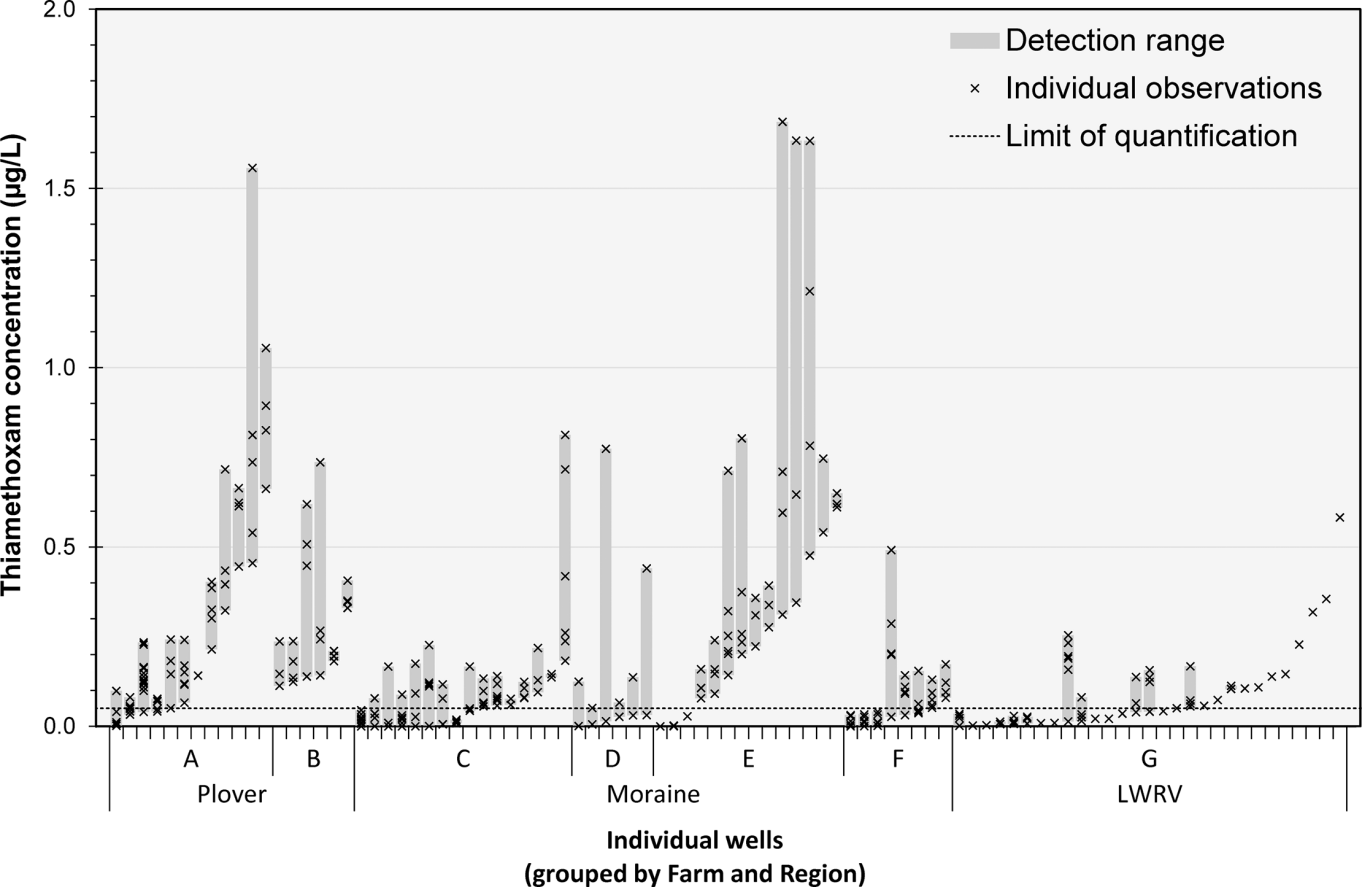
TMX concentration ranges for each high capacity well, 2013–2015. High-capacity wells are grouped by farm, arranged in order of increasing minimum TMX concentration. Vertical bars indicate concentration range (minimum to maximum) observed in each well, with individual observations represented as crosses. The limit of quantification of the analytical kits (0.05 μg/L) is indicated as a dashed horizontal line. In this text, values above this line are referred to as positive detections.

An analysis of detection ranges within individual wells confirms the results of our variance component analysis which identified individual well as the most significant explanatory factor among spatial factors. While we did find variation in mean TMX detection between farms, we also observed a high degree of variation in detections in individual wells at different time points (**Fig 5**). In the 47 wells we sampled that had more than two positive detections (out of a total of 91 wells), the detection range was an average of 0.26 ppb, and in all four wells where the highest TMX detections were observed (>1.5 μg/L), we also observed detections below 0.5 μg/L. In fact, higher average detection in these resampled wells was strongly correlated with a wider range of detections at those same wells (R^2^ = 0.65).

## Discussion

In this study, we noted no obvious long-term rising or falling trends in detection levels in this study (**Fig 3**). Within our weekly sampling data, we did note a pattern of rising and falling concentrations over the course of a single growing season (**Fig 4**), so even though we observed no trends in detection levels across years, contamination profiles do vary at individual wells within a season (**Fig 5**). This high degree of variability in detections strongly suggests that neonicotinoid contaminants are transient in these systems and a more frequent sampling regimen at additional wells would clarify some of the temporal dynamics at play. In addition, it has been reported that at least in surface water systems, grab sampling can result in variable contaminant detections that under-represent average contamination levels by 50% and maximum concentrations by 1 to 3 orders of magnitude [35].

We suspect that contaminant detections in groundwater samples taken from high-capacity wells are less variable than samples taken from static test wells or surface water grab samples because of the larger, deeper sources of water and non-point contamination sources, but in future work we would consider taking several samples at each well over a short period of time to potentially reduce variability. Despite high variance in TMX concentrations, certain farms, and certain individual wells within those farms, were more likely to have repeated above-average TMX concentrations in well water samples than others (**Fig 5**). We are currently working on developing explanatory models for neonicotinoid concentrations reported in this paper based on factors such as cropping history in the vicinity of each well, physical well parameters, and hydrological factors including water table depth and proximity to watershed borders. We hope that identifying specific explanatory factors contributing to higher levels of groundwater contamination can help build a more complete picture of the processes and risk factors associated with the use of neonicotinoid insecticides in an agricultural context.

We detected TMX in high-capacity irrigation wells at concentrations (<0.05–1.67 μg/L; **Table 3**) similar to, if slightly lower, than those reported in the same region by the Wisconsin DATCP over the same time period (<0.05–3.89 μg/L; **Table 2**). This difference in concentration levels could be partially explained by the different sources of water: in this study, we drew water from high-capacity irrigation wells, which are usually installed with intake screens relatively deep within the aquifer (**Table 1**), while the Wisconsin DATCP drew water samples from shallow groundwater monitoring wells. Deeper sources of water would likely be less contaminated as they are farther from surface sources of neonicotinoid contaminants [36]. Mean TMX detection from our survey of high-capacity irrigation wells in Wisconsin (0.28 μg/L) is within the range of previous reports of environmental detections of TMX near agricultural fields or in watersheds where these compounds are typically applied: a maximum TMX concentration of 0.032 μg/L from shallow groundwater and 0.376 μg/L from surface water runoff was reported from sites in Iowa where no seed treatments had been applied for two years [37]; maximum TMX concentrations of 0.46 μg/L from field-edge runoff and 0.16 μg/L from subsurface tile drains in agricultural fields were reported in Canada [38]; TMX detections up to 0.075 μg/L were reported from stream water in Ohio [15] and up to 0.185 μg/L in stream water in Iowa [16]; TMX concentrations up to 0.355 μg/L were reported from snowmelt in Canada [13]; and up to 1.49 μg/L was reported from wetlands adjacent to cultivated fields in Canada [5]. Previous work in central Wisconsin has also noted TMX detections up to 8.93 μg/L in shallow groundwater test wells and up to 0.580 μg/L from a small number of irrigation wells [2].

In addition to these environmental detections, several studies including this paper have reported detections of neonicotinoids in drinking water. Here we reported TMX detections in private potable wells in central Wisconsin at concentrations up to 1.43 μg/L, IMD detections up to 1.59 μg/L, and CLO detections up to 3.88 μg/L (**Table 2**). In the Mekong Delta in Vietnam, TMX, among multiple other agrochemicals, was reported in groundwater and surface water used as sources of drinking water for the local populations [39]. Worryingly, a recent study in Iowa detected neonicotinoids in treated municipal drinking water suggesting conventional water treatment practices are unable to effectively screen out these contaminants [17]. Neonicotinoids are not currently considered a human health hazard at concentrations now being reported [40] but the frequency of detections reported in surface water, groundwater, and treated drinking water is worth noting.

While most previous neonicotinoid surveys have focused on surface water contamination or direct field-edge runoff, we reported frequent detections of these compounds in both shallow and deep groundwater samples (from monitoring wells and irrigation wells, respectively). Depending on local hydrology, up to 90% of streamflow may be attributed to groundwater discharge [36]. In intensive agroecosystems such as the US Midwest, stream water has been found to contain dozens of contaminants, including fungicides, herbicides, insecticides, and their degradates [41,42]. In central Wisconsin specifically, the geology is characterized by highly permeable glacial deposits with shallow, mobile groundwater [43,44], so we expect that detections of neonicotinoids in groundwater will translate into detections in nearby surface water systems, and preliminary investigations in our lab confirm this. All of the watersheds where well samples were collected for this study have high fractions of agricultural land use (28–50%; USDA NASS Cropland Data Layer, https://nassgeodata.gmu.edu/), reducing groundwater recharge routes that do not pass through a cultivated field surface, leaching agricultural contaminants into groundwater and subsequently into adjacent surface waters. Aquatic invertebrates living in these streams are highly sensitive to neonicotinoid insecticides and form a critical link in aquatic food chains [6,45]. Any reduction of aquatic invertebrate populations would be felt throughout local food chains resulting in reduced ecosystem health, biodiversity, and recreational opportunities [1,3,6,46,47].

One of the initial aims of this study was to quantify the spatial distribution of neonicotinoid contamination within Wisconsin’s Central Sands as prior detections had only been reported from a small number of wells spread widely across the region [2,30,31]. We found significant variations in TMX detections between farms (generally separated by 5–20 km), and that even adjacent wells separated by less than 1 km could vary in detected TMX concentrations by over one order of magnitude (**Figs 2 and 3**), suggesting neonicotinoid applications at individual fields may be responsible for elevated detections at nearby wells, rather than a more uniform detection profile indicative of a higher degree of groundwater mixing and dilution.

While concentrations varied from well to well, detection rates of TMX were overall relatively high in our study area, a finding confirmed by DATCP’s parallel groundwater monitoring activities (**Fig 1**), and suggesting that in the aggregate, agricultural activities in central Wisconsin are contributing to widespread detections of neonicotinoids in both deep and shallow groundwater. Considering that DATCP has tested samples from across the entire state, but detections were primarily reported in central Wisconsin, we suspect that there is an intersection of neonicotinoid usage rates and contamination risk factors in this part of the state that contribute to these frequent detections. While this study focused on central Wisconsin specifically, the intersection of neonicotinoid usage and groundwater systems vulnerable to contamination from agricultural activities covers a large part of the Midwestern United States and Great Lakes region and elsewhere [15,41].

## Conclusions

The frequency of thiamethoxam detections in both shallow and deep wells throughout the study region underscores the need for growers to be judicious in the use of these chemicals when operating in areas at elevated risk of groundwater contamination. In combination, frequent neonicotinoid detections in shallow field-edge monitoring wells, deeper high-capacity irrigation wells, and private potable wells highlight the potential risk of agricultural contaminants to appear throughout an entire aquifer underlying an intensive agroecosystem. Neonicotinoids are popular and effective insecticides whose usage will likely continue to expand, absent new regulatory action or the commercialization of next-generation insecticides. Their usage is not currently considered a human health hazard, but it is becoming increasingly clear that neonicotinoids are easily mobilized into the environment after field applications [6,48]. Evidence is also mounting that even very low environmental concentrations of neonicotinoids are harmful to aquatic and terrestrial invertebrates and damage local ecosystems [47]. Clearly, alternative cultural or chemical pest control strategies must be implemented to reduce neonicotinoid-related environmental impacts [49]. We hope that additional studies on groundwater contamination are pursued in other at-risk areas to expand our understanding of water quality issues related to intensive agriculture.
